# Packing and flow performance of binary adhesive mixtures for inhalation of different drug loads and their relationships to aerosolisation

**DOI:** 10.1016/j.ijpx.2025.100358

**Published:** 2025-07-17

**Authors:** Anna Simonsson, Tobias Bramer, Alex Wimbush, Göran Alderborn

**Affiliations:** a Department of Pharmaceutical Biosciences and the Swedish Drug Delivery Center, Uppsala University, Box 591, SE-751 24 Uppsala, Sweden; b Inhalation Product Development, Pharmaceutical Technology & Development, AstraZeneca Gothenburg, Sweden

**Keywords:** Binary mixtures, Inhalation, Mechanical properties, Dispersion properties, Blend architecture, Flowability-dispersibility relationships

## Abstract

The aim of this study was twofold. First, to examine the mechanical properties (packing and flow) of a series of adhesive mixtures, consisting of two different lactose carriers and varying concentrations of budesonide, using a range of test methods. Second, to investigate if any of the test methods correlate with the dispersibility of the mixtures, i.e. the fine particle fraction and mass median aerodynamic diameter. The mechanical properties assessed included packing, shearing, permeability and compressibility. Dispersion data were generated using an impactor operated at two pressure drops (0.5 and 4 kPa). To explore correlations between the mixture properties, Principal Component Analysis and Pearson correlation were used as statistical tools.

The different test methods yielded different property-drug load relationships, which can be classified into two groups: First, packing density and shearing properties, and second, permeability and compressibility. The methods in the first group produced markedly fluctuating property-drug load relationships, characterised by two distinct waves. This type of property-drug load relationship was similar to that observed in the dispersion experiments, and significant correlations were found between shearing properties and dispersibility. Thus, any correlations between mechanical and dispersion properties depend on the choice of the test method used. The underlying cause of this co-variation is the parallel effect of both the blend architecture and the structure of the adhesion layer on mechanical and dispersion properties.

## Highlights

• Packing and flow methods could be classified into two groups.
• Correlation between mechanical properties and dispersibility depended on methods.
• Dispersibility and shear properties had a similar dependency of blend architecture.
• Permeability correlated to pore diameter but not to dispersibility.
• Blend state is critical for several performance aspects of adhesive blends.

## Graphical abstract

**Figure:**
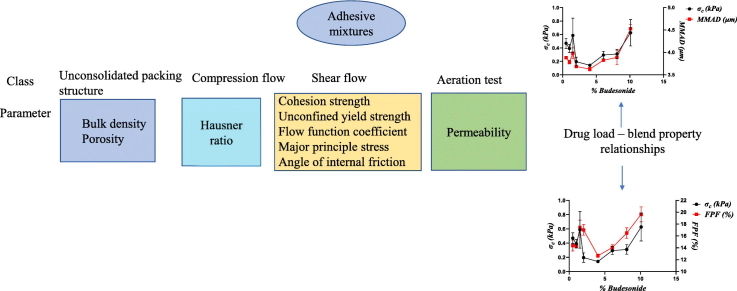

## List of symbols

**Table:** 

Symbol	Meaning	Unit
*ff*_c_	Flow function coefficient (flow index)	–
*h*_0_	Unconsolidated powder bed height	m
*h*_30_	Consolidated powder bed height	m
*HR*	Hausner ratio	–
*ΔP*	Pressure drop	N/m^2^
*k*	Unconsolidated powder permeability	m^4^/Ns
*u*	Air velocity	m/s
*θ*	Pore diameter	m
*η*	Air viscosity	Ns/m^2^
*ε*	Unconsolidated powder porosity	%
*ρ*_b_	Unconsolidated powder bulk density	kg/m^3^
*ρ*_p_	Particle gas pycnometry density	kg/m^3^
*σ*_*c*_	Unconfined yield strength	N/m^2^
*σ*_1_	Major principal stress	N/m^2^
*S*_*v*_	Volume specific surface area	m^−1^
*τ*_0_	Cohesion strength	N/m^2^
*φ*	Angle of internal friction	Degrees

## 1 Introduction

Dry powder inhaler (DPI) formulations are often prepared as adhesive mixtures, typically consisting of two or three components, i.e. a drug, a carrier and optionally, a dispersion facilitating agent. The adhesive units (carriers with drug particles adhered to their surfaces) form spontaneously when the carrier and drug particles are agitated. However, despite seemingly simple powder mixtures, the aerosolisation propensity of adhesive blends may exhibit a complicated dependency on the concentration of the active pharmaceutical ingredient, i.e. the drug load (Simonsson et al., 2024). A proposed explanation for this complex relationship (Simonsson et al., 2024) is that the aerosolization performance is controlled by the blend architecture, specifically, whether the drug particles are bonded or non-bonded to the carrier surface, and the micro-structure of the bonded adhesive layer. To conceptualise the evolution of the spatial distribution of drug particles with drug load, a classification system denoted as the blend state theory has been proposed (Rudén et al., 2019). The change in blend state with drug load (from blend state S1a to S2b) corresponds to a shift in the localisation of the bonded drug particles and the micro-structure of the adhesion layer, i.e. towards a thicker and more uneven layer.

Rudén et al. (2021) earlier reported that an increased drug load (i.e. consecutive blend states) also affected the flowability, as assessed by the Hausner ratio, of adhesive mixtures. Since such mechanical properties of a powder are critical for transport into and packing in small containers, they may also affect the filling of a DPI and the dosing during use of multiple dose DPIs. In other words, the blend state may be relevant for manufacturing, dosing and aerosolisation during use of adhesive blends. A special aspect addressed in the literature in this context is whether indications of mechanical properties could be used to predict and optimise the dispersion performance of an adhesive blend. This problem was first raised by Concessio et al. (1999), who hypothesized that numerical descriptors of the flowability of carrier powders predict the detachment and dispersion of fine drug particles. The authors studied six formulations of three carriers with the same sieve cuts and reported a positive (direct) relationship between increased carrier powder flowability and an increase in fine particle mass. Following this paper, a series of studies have been published (Louey et al., 2003; Jones et al., 2010; Le et al., 2010; Cordts and Steckel, 2025; Kinnunen et al., 2014; Hertel et al., 2018; Shalash et al., 2018; Sun et al., 2020; Rudén et al., 2021; Almansour et al., 2022; Stankovic-Brandl et al., 2022) on the relationship between mechanical or rheological powder properties and the dispersion performance of adhesive mixtures. The reported findings can be summarised as follows: First, regarding ternary adhesive mixtures with different concentrations of micronised fines (usually lactose), good direct correlations have been observed for cohesiveness-dispersion relationships. Typical for these studies is the use of a low drug load (less than 5 %). These relationships are typically inverted, i.e. a reduced flowability (increased cohesiveness) and a more closed pore structure of the mixture facilitate dispersion. Both uni-phasic and bi-phasic relationships have been reported; for the latter, the phase transition has been associated with the formation of self-agglomerates. Secondly, regarding binary mixtures with carriers of different materials and a low drug load, a direct correlation between the degree of dispersion and the powder flow properties have been reported, i.e. powders showing good flow properties (low cohesion) dispersed well during aerosolisation. Thirdly, regarding binary mixtures prepared of a broad range of drug loads, the general trend reported is that an increased drug load results in an increased cohesiveness and enhanced dispersion. It is evident that considerable variations in the experimental conditions used exist between the studies, affecting the possibility to draw generalised conclusions.

Considering the importance of packing and flow performance of adhesive mixtures, there is scant information in the literature on the effect of drug concentration on both the mechanical properties and aerosolisation performance of binary adhesive mixtures over a wide range of drug loads. This is an issue that warrants closer inspection. Since mechanical properties can be assessed using different methods, the resulting mechanical property-drug load relationships may depend on the choice of the test method used. Moreover, whether any of these changes show co-variation with the evolution of dispersibility with drug load may consequently depend on the choice of test method. In this investigation, we study the mechanical properties of the same series of adhesive mixtures used earlier to investigate aerosolisation-drug load relationships (Simonsson et al., 2024). We first ask whether different methods typically used to assess the mechanical properties of adhesive blends give similar or different changes in derived measures with drug load, and then we ask whether any of these methods show changes with a pattern similar to the evolution in blend dispersibility with drug load.

## 2 Materials and methods

### 2.1 Materials

Binary adhesive mixtures were prepared using micronised Budesonide, provided by AstraZeneca Gothenburg (Sweden), and the carriers Inhalac 70 and Inhalac 230 (Meggle, Germany). Some particle characteristics of these three powders have been reported elsewhere (Simonsson et al., 2024). Budesonide had a median particle size of 2.1 μm, while the carriers Inhalac 70 and Inhalac 230 had median particle sizes of 247 μm and 104 μm, respectively.

The composition, preparation and characterisation of homogeneity and aerosolisation properties of the adhesive mixtures used in this study are reported elsewhere (Simonsson et al., 2024). All powders and adhesive mixtures were stored in a climate-controlled room at a temperature of 21–24 °C and a relative humidity of 30–34 % for a minimum of 24 h before characterisation.

### 2.2 Powder packing

#### 2.2.1 Packing density of unconsolidated powder

The apparent particle density (*ρ*_p_) was measured by Helium pycnometry (AccuPyc 1330, Micrometrics Instruments, Nordcross, USA). The steel cylinder (with a volume of 11.38 cm^3^) was filled with a powder sample to approximately 50 % of its volume. The sample weight recorded, and the powder volume of each sample was determined by five consecutive measurements, which took approximately 10 min in total. Reported values are the mean and standard deviations of the three samples.

The unconsolidated powder bulk density (*ρ*_b_) was measured by powder pycnometry using a sample steel cylinder of 20.05 ml, manufactured by AstraZeneca Gothenburg (Mölndal, Sweden), as reported earlier (Rudén et al., 2018). As an indication of the packing density of the powders, the unconsolidated powder porosity (*ε*) was calculated as:

(1) $$\varepsilon =1-\left(\frac{\rho_{b}}{\rho_{p}}\right)$$

Reported values are the mean and standard deviation of the three measurements.

#### 2.2.2 Permeability of unconsolidated powder

The permeability of unconsolidated powders *(k*) was determined using a steady-state permeameter. Approximately 2 g of powder was scooped into a graduated cylinder with a diameter of 11.42 mm, giving a powder bed height of about 25 mm. The weight and height of the powder bed were thereafter determined. The cylinder was connected to the permeameter, and air was pumped through the powder bed at a series of flow rates (10–50 ml/min), which were recorded by a flowmeter (Brooks 5850E, Brooks Instruments B.V., the Netherlands). The resultant pressure drop across the bed of powder was simultaneously recorded by a manometer (Micromanometer P200 S, Digitron Instrumentation Ltd., UK). From the slope of the linear relationship between air velocity (*u*, in the range of 2–8 mm/s) and pressure drop (*ΔP*) over bed height (*h*), the unconsolidated powder permeability (*k*) was calculated as follows:

(2) $$k=\frac{uh_{0}}{\mathrm{\Delta P}}$$

Reported values are the mean and standard deviation of the three measurements.

The pore diameter (θ) was calculated according to the following equation:

(3) $$\theta ={\left(\frac{k80\eta }{\varepsilon }\right)}^{1∕2}$$

The definition of permeability *k* of the powder bed used here is $k=\frac{u}{x}$, where the air velocity is $u=\frac{v}{\mathrm{At}}$ and the pressure drop over bed height is $x=\frac{\mathrm{\Delta P}}{L}$. The diameter of the pores in the powder bed (θ) can be calculated as (Orr and Dallavalle, 1959):

(4) $$\theta =\left(\frac{4}{s_{v}}\right)\cdot \left(\frac{\varepsilon }{\left(1-\varepsilon \right)}\right)$$

Where S_v_ is the volume-specific surface area and ε is the porosity if the powder bed.

Assuming laminar mass flow of air through the column, S_v_ can be calculated by the Kozeny-Carman equation, i.e.:

(5) $$\;s_{v}^{2}=\left(\frac{x}{5\mathrm{u\eta}}\right)\cdot \left(\frac{\varepsilon^{3}}{{\left(1-\varepsilon \right)}^{2}}\right)$$

Where η is the viscosity of air (1.8*10^−5^Ns/m^2^).

By combining eqs. (4), (5), the expression for the calculated pore diameter is generated as in eq. 3.

### 2.3 Powder compressibility

As an indication of the compressibility of the powders, the Hausner ratio (*HR*) was calculated as the ratio between the height of the powder bed prior to compression (*h*_0_) and the height of the powder bed at a compression stress of 30 kPa (*h*_30_), (both generated using a Freeman FT4 powder rheometer (Freeman Technology, Tewkesbury, UK)) i.e.:

(6) $$\mathrm{HR}=\left(\frac{h_{0}}{h_{30}}\right)$$

Samples of approximately 10 g were initially put into a 10 ml borosilicate cylinder, with a cross-section diameter of 25 mm. The samples were initially gently conditioned with a conditioning blade, which was then changed to a ventilated piston, compressing the sample with a normal force ranging from 1 to 30 kPa. During compression, a constant airflow (2 mm/s) was maintained through a perforated metal base plate at the bottom of the cylinder. The recorded powder bed heights prior to compression and after compression at 30 kPa were then used to calculate *HR* (eq. 6).

The reported values are the mean and standard deviation of the three measurements.

### 2.4 Shearing properties

The shearing properties of powder samples were assessed using the rotational shear head of the Freeman FT4 powder rheometer (Freeman Technology, Tewkesbury, UK), with a diameter of 24 mm and equipped with 18 vertical blades. The measurement procedure consisted of the following steps: Conditioning, consolidation, pre-shearing and shearing. The powder was filled into a 10 ml sample cup, after which the powder was conditioned by a rotating helical blade that was moved downwards and upwards in the shear cell. Thereafter, an initial consolidation stress of 3 kPa was applied to the powder by a ventilated piston. The ventilated piston was then replaced with the shear blade, and a pre-defined normal stress of 2 kPa was applied. The powder was then pre-sheared by rotating the piston at a rate of 18^o^/min until a steady state was obtained, and the shear stress and normal stress were recorded as the pre-shear point. Once the pre-shear point was defined, the normal stress was decreased, and the sample was once again sheared to obtain a yield point. Five normal stresses below the pre-shear point, at decreasing loads (between 2 and 1 kPa), were used to obtain a yield locus, i.e. each test resulted in one pre-shear point and five yield points. The pre-shear procedure was repeated between each yield point experiment.

A linearized yield locus was obtained by linear regression of the yield points. From the linearized yield locus, two shear parameters were calculated: (1) The cohesion strength (*τ*_0_), i.e. the intercept of the extrapolated yield locus on the shear stress axis; (2) The angle of internal friction *(φ)*, i.e. the angle between the yield locus and the normal stress axis.

Using Mohrs circles analysis, four shear parameters were calculated: (1) The unconfined yield strength *(σ*_*c*_*)*, i.e. the intercept at the normal stress axis of the Mohr circle passing through the origin and tangent to the yield locus; (2) The major principle stress *(σ*_1_), i.e. the intercept at the normal stress axis of the Mohr circle passing through the pre-shear point and tangent to the yield locus; (3) The flow function coefficient *(ff*_c_, also denoted the flowability index), i.e. the ratio between the major principal stress and the unconfined yield strength.

The parameters were obtained using the data analysis software (version 4.1) provided by Freeman technology.

### 2.5 Statistical analysis

Reported data in tables and figures are mean values and standard deviations based on three independent observations, unless otherwise stated.

To identify patterns and groupings in the data set of the different variables related to the packing and flow properties of the mixtures, principal component analysis (PCA) was performed using the PCA application in the software GraphPad Prism 9.2. To ensure that the variables being analysed were all on the same measurement scales, the data were initially normalised so that each variable had a mean of zero and a standard deviation of one.

As a means to derive correlations between different values, Pearson correlation analysis was performed using an application in GraphPad Prism 9.2.0. The Pearson correlation displays the direction and significance of a potentially linear relationship between the two data sets. Correlation matrices were first calculated for the packing and flow data, and secondly for the packing, flow and aerosolisation data (in the latter case, the carrier only mixtures were obviously excluded, as well as the highest drug loads for the respective carrier). Significant linear correlations between variables were assumed when *P* < 0.01 (packing and flow data) and *P* < 0.05 (for packing, flow and aerosolisation data).

## 3 Results and discussion

### 3.1 Experimental design

In this paper, some mechanical properties (packing and flow properties) of drug-carrier adhesive mixtures are studied, and we investigate how the drug load affects these properties. The aerosolisation performance of the same series of adhesive blends has been reported earlier (Simonsson et al., 2024), and we also explore if any indications of packing and flow properties correlate with their in vitro aerosolisation performance. In the aerosolisation study, a Next Generation Impactor (Copely Scientific, UK) equipped with a laboratory inhaler device (a Screenhaler coupled with a Turbuhaler mouthpiece) of a simple design (Thalberg et al., 2016) was used. Two pressure drops were used during the impactor experiments, i.e. 0.5 and 4 kPa. The higher pressure drop, i.e. 4 kPa, was chosen since it is often used in dispersion experiments using an NGI (Stankovic-Brandl et al., 2022; Almansour et al., 2022). In order to assess if the pressure drop markedly affects the studied relationships also a considerably lower pressure drop (0.5 kPa) was used. It is acknowledged that this is a very low pressure drop lower than what is typically generated by the patient during inhalation using a commercial device (Clark et al., 2020) The reason for choosing this very low pressure drop was to assess if the interplay between packing and flow properties of the powders and their aerosolization is dependent on the pressure drop during actuation.

The mixtures were manually poured into the inhaler device, forming an unconsolidated bed or cone of powder before being exposed to flowing air.

In order to derive indications of the flow properties of powders, a multitude of tests have been developed and are used in different scientific areas, such as pharmaceutics, metallurgy and polymer science. Attempts to group the tests into classes have also been reported, e.g. by Marchetti and Hulme-Smith (2021). The tests are based on different types of powder flow, such as gravitational and compression flow. For adhesive mixtures, the mixture's behaviour under aerated conditions, i.e. when air flows through the powder, has also been used as a means to characterise mechanical properties of adhesive mixtures. The reason behind the use of aerated conditions is to derive information that may be relevant for the sequence of events involved in the dispersion of the drug, where air entrainment of the power bed is often considered the first step (Shur et al., 2008).

**Fig. 1:**
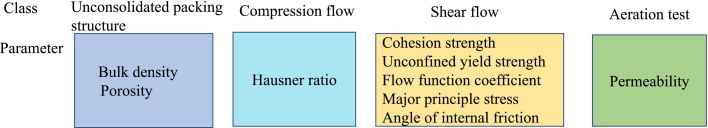
Overview of test methods used to investigate packing and flow performance, grouped into the chosen classes.

A broad range of tests have been used to mechanical properties of adhesive mixtures. The tests can be divided into six classes depending on the type of handling of the powder, as follows: (1) Packing in an unconsolidated or consolidated state (such as bulk density); (2) Compression (such as the Hausner ratio); (3) Shearing (such as cohesion strength); (4) Aeration (such as permeability); (5) Rheology; and (6) Gravity driven tests, such as static or dynamic angle of repose and flow time. In this study, one or more tests from each of the first four classes were used to broadly characterise the mechanical properties of the adhesive blends (Fig. 1). These tests provide indications of powder properties in terms of packing state and its behaviour while subjected to two types of stresses, i.e. compression and shearing. Packing was assessed in an unconsolidated state since the mixture dispersed into the inhaler used in this study was considered to be unconsolidated. The objective was to select tests that provide data with low variability and good discrimination (Leturia et al., 2014; Rudén et al., 2018), yielding parameters that are, in relative terms, physically less complex (Leturia et al., 2014). Compression and shear tests were considered important because metrics derived from such tests have been used to classify the flow performance of powders using qualitative descriptions based on the test scores. Finally, permeability was deemed important, as it may be critical for the air entrainment of the powder in the inhaler (Shalash et al., 2018; Almansour et al., 2022).

Regarding group 5, the rheological tests provide indications of the resistance to the motion of a rotating blade in a powder bed. In the Freeman FT4 rheometer, the derived rheological metrics are referred to as the specific energy (SE) and the normalised basic flowability energy (NBFE). In earlier studies (Rudén et al., 2018; Almansour et al., 2022), it was reported that these measures had a limited ability to discriminate between adhesive mixtures with different payloads. Thus, these rheological tests are not used in this study. Gravity-driven tests (group 6) were also excluded due to the need for large quantities of powder for the measurements, which were not available for this study.

To group the parameters obtained from the tests, two statistical evaluations were performed: principal component analysis and Pearson correlations. The PCA was mainly performed to visualise patterns, similarities and differences among the various parameters. Thereafter, the effect of drug load on the evolution of the correlated parameters was compared. Finally, the indications of packing and flow were compared to the dispersibility (assessed by FPF and MMAD) – and the drug load relationships reported earlier (Simonsson et al., 2024).

### 3.2 Correlations between packing and flow performance

#### 3.2.1 Inhalac 70

**Fig. 2:**
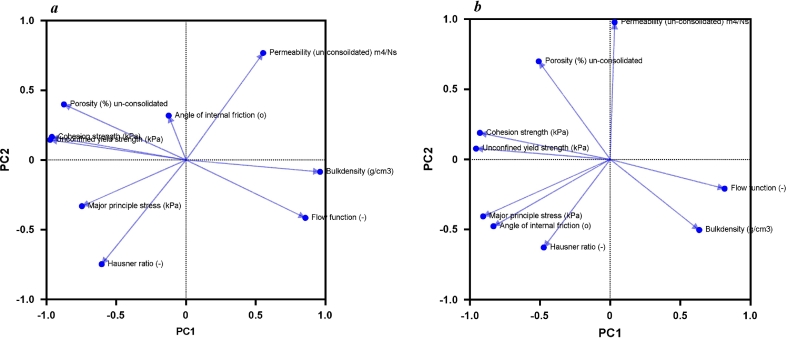
PCA plots of derived parameters of packing and flow performance for all mixtures of Inhalac 70 (a) and Inhalac 230 (b).

A principal component analysis (PCA) was used to identify similarities or co-variations among the metrics obtained from the packing and flow tests (Fig. 2). Regarding variables *within the same test class,* unsettled bulk density and porosity showed a negative co-variation, i.e. increased porosity correlated with decreased bulk density (an inverse correlation). Porosity is a more refined measure of packing structure than bulk density since it takes into account the mean particle density of the mixtures. However, a co-variation is expected for these mixtures due to the minimal difference in particle density between the carrier and the drug particles. For the variables obtained through shear testing, cohesion strength, unconfined yield strength and the flow function coefficient were grouped together. Previous studies (Wang et al., 2016; Sun and Sun, 2019) suggest that cohesion strength and unconfined yield strength are expected to correlate, thus indicating the same shearing properties of the powder. In contrast, the major principle stress did not show the same strong co-variation, and the angle of internal friction showed low loadings for both PC1 and PC2. In addition, none of these parameters correlated significantly (*P* < 0.01) with any of the other parameters when performing the Pearson correlation analysis. The angle of internal friction reflects the extent to which the shear stress changes with a given change in normal stress, making it a unique descriptor of the shearing performance of the Inhalac 70 blends.

In terms of variables *from different test classes,* the PCA plot revealed two distinct groups of data showing co-variation. First, the unconsolidated permeability and the Hausner ratio and secondly, the bulk density (or porosity) and the shearing variables: cohesion strength, unconfined yield strength and the flow function coefficient.

**Fig. 3:**
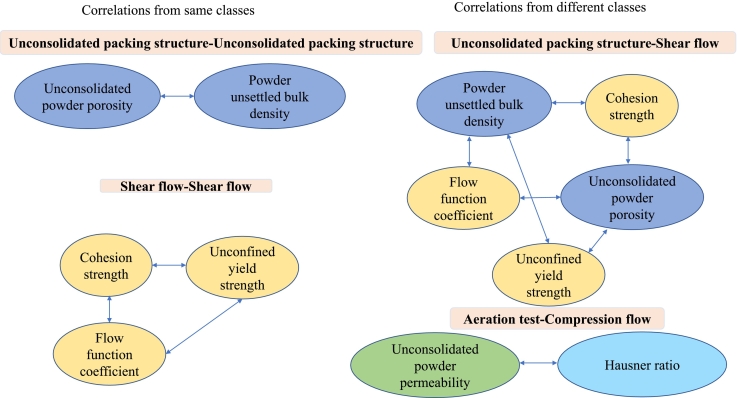
Correlations from the Pearson analysis of properties (P < 0.01) within the same class of tests and across different classes of tests for all mixtures of Inhalac 70.

For the Pearson correlations, an overview of significant correlations (P < 0.01) is presented in Fig. 3. The data is based on linear correlations between two variables, but the underlying relationships between a given variable and the drug load may not be strictly linear. The outcome of the Pearson analysis (PA) compared favourably to the PCA, providing a more detailed description of correlations and their subsequent significance. Regarding correlations of metrics *from the same test class*, the measures of the unsettled bulk density and porosity showed a significant negative correlation. Moreover, cohesion strength, unconfined yield strength and the flow function coefficient obtained from the shear tests were also significantly correlated. Regarding variables obtained through tests *from different classes* (Fig. 3), significant correlations were observed between bulk density, porosity, cohesion strength, unconfined yield strength and the flow function coefficient. Additionally, a significant correlation was identified between permeability and the Hausner ratio.

**Fig. 4:**
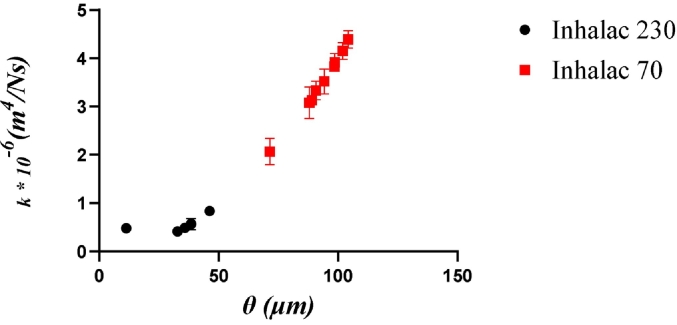
The effect of pore diameter on unconsolidated permeability for all mixtures of Inhalac 230 and Inhalac 70.

Neither the PCA nor the PA showed any co-variation between the packing density of the unconsolidated mixtures, as assessed by bulk density and porosity, and their permeability. While permeability is a function of the porosity of the bed, it also depends on the diameter of the pores through which the air flows. In Fig. 4, permeability is plotted as a function of pore diameter, calculated using eq. 3. For both carriers, the permeability related in a non-linear way to the pore diameter, i.e. both carriers gave a similar type of relationship between these variables.

Thus, the permeability was directly related to the separation distance between the adhesive units forming the mixture. The pore diameter appeared to be a critical factor for the compressibility of the mixtures, while the shearing behaviour was predominantly related to the mixture's porosity.

#### 3.2.2 Inhalac 230

**Fig. 5:**
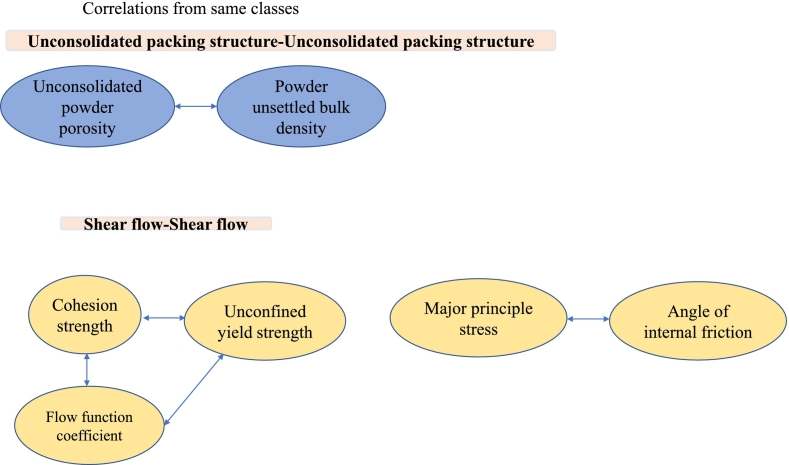
Correlations from the Pearson analysis (*P* < 0.01) of properties within the same class for all mixtures of Inhalac 230.

The number of Inhalac 230 mixtures studied was smaller than that of Inhalac 70 due to the lower loading capacity of the former. This difference in loading capacity is probably related to the differences in particle mass and morphology between the carriers. The low number of mixtures was probably a limiting aspect regarding the possibility of identifying co-variations between variables obtained from the packing and flow tests. Nevertheless, the PCA showed similar groupings of variables to those observed for Inhalac 70. Regarding the PA, some significant correlations were obtained within the same class of test methods, similar to the findings for Inhalac 70 (Fig. 5). However, no significant correlations were observed for variables from different test classes. For the Inhalac 230 mixtures, the major principle stress (MPS) and the angle of internal friction (AIF) showed a similar progression with drug load giving, according to the Pearson analysis, a good correlation between these measures of shear properties. For both measures, an initial valley at 1 % drug load was obtained followed by a gradual increase. The trend was that an increased AIF, i.e. a larger increase in shear stress with normal stress, correlated to an increased MPS. For the Inhalac 70 mixtures, a correlation between these measures was however not obtained probably due to that the AIF showed a more fluctuating pattern with drug load up to 2 % than the MPS. However, above 2 % drug load, both measures showed a similar and gradual increase with the same trend as for Inhalac 230.

### 3.3 Effect of drug load on evolution of packing and flow properties

#### 3.3.1 Inhalac 70

**Fig. 6:**
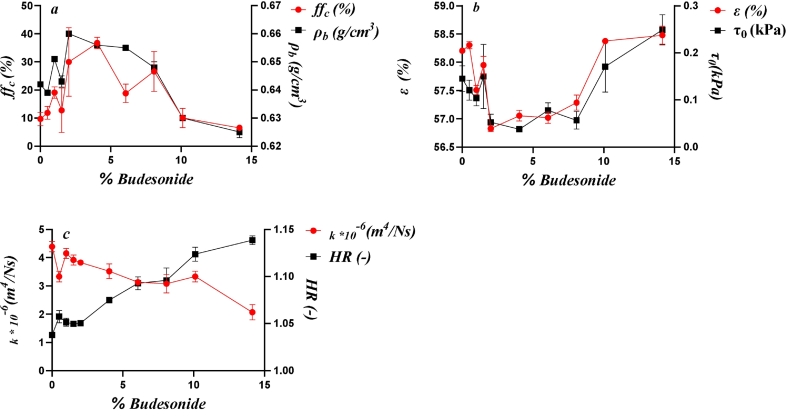
The effect of drug load on the evolution of packing and flow parameters, showing similarity according to the PCA and significant correlations according to the Pearson analysis for all mixtures of Inhalac 70. Mean values and standard deviations (n = 3). (a) Flow function coefficient and bulk density. (b) Unconsolidated porosity and cohesion strength. (c) Unconsolidated permeability Hausner ratio.

The statistical analyses indicated correlations between several variables related to the packing and flow performance of the mixtures. Fig. 6 shows the relationships between drug load and the variables that correlated according to the PCA and the PA for the Inhalac 70 mixtures.

The bulk porosity decreased with the addition of small amounts of drug up to 1 %, thereafter increased, and then decreased markedly after admixing of 2 % drug. In the drug load range of 2–8 %, the porosity remained nearly constant. However, beyond this range, porosity increased markedly and approached a constant value at 10 % and 14 % drug content. Thus, these fluctuations can be summarised as two distinct valleys, with the first covering a restricted drug load range and the second spanning a larger range. The same pattern was observed for bulk density, but in the opposite direction. Both the flow function coefficient and the cohesion strength displayed similar types of fluctuations. The addition of fine drug particles to the carrier resulted in a reduction in powder cohesion compared to the carrier powder alone until a drug load of 8 % was reached. Thereafter, when blend state 3 was entered the powder cohesion increased.

For permeability and the Hausner ratio, the property-drug load relationships were somewhat less complex. The initial addition of the drug resulted in a decrease in permeability, followed by an increase, after which permeability decreased continuously with increasing drug load. A similar effect of drug load was observed for the Hausner ratio, with a dominating trend towards an increased ratio with drug load. Thus, the addition of fine drug particles to the carrier was generally characterised by a decrease in both permeability and flowability (as of HR). Thus, depending on the test method used, apparently conflicting effects of fine drug addition on mixture flowability were observed.

In summary, one can distinguish between two types of progression patterns in the property-drug load relationships reported in Fig. 6. The first one is characterised by marked fluctuations with drug load – as shown for bulk density, porosity, cohesion strength and the flow function. The second pattern shows a more gradual change with drug load, evident in in the permeability and the Hausner ratio. Notably, no correlation was found between porosity and permeability, indicating no direct relationship between porosity and the pore diameter of these mixtures.

#### 3.3.2 Inhalac 230

**Fig. 7:**
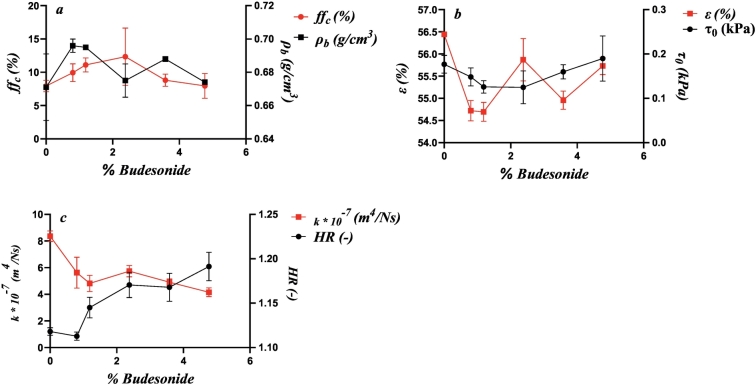
The effect of drug load on the evolution of packing and flow parameters, showing similarity according to the PCA and to the Pearson analysis for all mixtures of Inhalac 230. Mean values and standard deviations (n = 3). (a) Flow function coefficient and bulk density. (b) Cohesion strength and unconsolidated porosity. (c) Unconsolidated permeability and Hausner ratio.

Fig. 7 shows the relationships between drug load and the variables that showed similarity correlated according to the PCA and PA for the Inhalac 230 mixtures. Due to the restricted range of drug loads possible for this carrier-drug combination, the relationships were less complex. However, two distinct types of property-drug load relationships, similar to those observed for Inhalac 70, were evident. Hence, bulk density, porosity, cohesion strength and the flow function formed one group, while permeability and the Hausner ratio formed the other. Porosity and bulk density exhibited a fluctuating relationship with two valleys, similar to Inhalac 70. Cohesion strength showed a U-shaped pattern, and the flow function displayed a similar pattern but in the opposite direction. For permeability and the Hausner ratio, the lowest addition of drug resulted in a decreased permeability and a constant Hausner ratio. Thereafter, a nearly constant permeability, with a tendency to a decrease with drug load, was obtained while the Hausner ratio increased more or less continuously with drug load. Thus, as with Inhalac 230, the effect of drug load on flow performance differed between tests, indicating differences in shearing and compression behaviour, respectively.

#### 3.3.3 Mechanisms of action of adsorbed drug on packing and flow of adhesive mixtures

An adhesive blend is an assembly of particles consisting of adhesive units; in addition, free drug fines may be present depending on the drug load. An adhesive unit is an agglomerate consisting of a single carrier particle to which fine drug particles are adsorbed, forming an adhesion layer. The adhesion layer may, depending on its strength, be more or less susceptible to changes in morphology and packing density when exposed to external forces (Rudén et al., 2019). Consequently, the adhesion layer may undergo changes in the structure when subjected to forces during flowability assessment. The blend state model proposed earlier (Rudén et al., 2019) describes how the distribution of fine drug particles on the surface of the carrier particles changes with increasing drug concentration. This transition involves a change from a strongly adsorbed, immobile layer to a looser, more responsive layer. In this study, we assume that in blend states referred to as 1 and 2, i.e. where all fine particles in the blend are bonded to the carrier and free fine particles are almost completely absent, the adhesive unit behaves as a coherent agglomerate. This agglomerate controls the interaction between the units, and thus also the blend flow performance. The adhesion layer can be described as affecting the surface roughness of the agglomerate, which will consequently change with drug load due to the build-up of an adhesion layer with different structural characteristics. Therefore, blend flowability is partly dictated by the adhesion layer structure, which evolves with drug load.

At an initial low range of drug loads (up to 2 %), some fluctuations in packing density and cohesion strength were observed, i.e. fluctuations in measures representing the unconsolidated state of the powders. In an earlier paper (Rudén et al., 2019), we reported that when fine particles are located predominantly in the cavities of the carrier rather than on the surface of the enveloped carrier, an increase in bulk density occurs due to the added weight of the adhesion unit compared to the carrier particle, with only a limited effect on the enveloped volume. Similarly, a small addition (1 %) of the drug increased the bulk density and reduced the porosity of the mixture, thus resulting in the formation of blend state 1. The cohesion strength and flow factor were also affected by this small addition of fine drug. Thereafter, at a drug load of 1.5 %, an increase in porosity was observed, followed by a decrease at 2 %, with nearly parallel changes noted in the cohesion strength. In a previous study (Simonsson et al., 2024), we differentiated between blend states 1a and 1b, and it is proposed that the initial fluctuations in porosity and cohesion strength can be explained by the different structures of the adhesion layer in blend states 1a and 1b. It is proposed that when the carrier cavities are almost saturated with fine drug (state 1b, occurring at a drug load of 1.5 %), the sharp corners of the carrier, which would otherwise penetrate the cavities, are pushed away, increasing both porosity and cohesion. Thus, the initial fluctuations observed with an increasing drug load up to 1.5 % may be attributed to a combination of increased adhesion unit density (1a) and some reorientation of the adhesive units (1b).

At an initial low range of drug loads (up to 2–8 %), small changes in packing density and cohesion strength were observed. In this range of drug load, mixtures studied here were previously proposed (Simonsson et al., 2024) to be in blend state 2. In contrast to cohesion strength, the Hausner ratio showed a gradual increase with drug load. Thus, depending on the type of flow involved in the measurement of flow performance, i.e. compression flow or shear flow, different dependencies on drug load may be observed. Compression involves the sliding of particles against each other while they repack to a higher density. Shearing, on the other hand, also involves the sliding of particles against each other, but along a yield plane at a constant bulk volume. While the fundamental process controlling the flow is similar for both types of flow, they do not exhibit the same relationship with drug load. Therefore, it seems that the adhesion layer structure in a blend state 2 situation may affect the friction conditions in the blend differently, depending on the type of flow involved in the test.

For shear flow, the addition of a small amount of drug decreased the cohesion strength compared to the carrier powder alone, with the drug acting as a glidant. Glidants are typically sub-micron sized particulate solids that, when mixed with larger particles in small proportions, can facilitate powder flow. A proposed mechanism (Majerová et al., 2016; Tran et al., 2019) suggests that they will be positioned in-between the larger particles, increasing the separation distance between the carrier particles and reducing the intermolecular forces between them. Thus, in small proportions, the bonding between the larger carrier particles will control the cohesion, which is reduced by the adhered fines, thereby increasing flowability. This decreased bonding may also facilitate closer packing of the adhesive units when poured into a container, resulting in decreased unconsolidated porosity. At high drug concentrations, approaching the maximum drug load, cohesion strength increases, and the fine drug hence acts as an anti-glidant. The formation of a thick enveloping layer of fine particles on the carrier surface reduces the pore diameter between the adhesive units, and layer-layer interlocking may prevent sliding ultimately increasing cohesion strength.

In a compression test, the adhesion units are forced into closer proximity. A consequence may be that the interactions between the adhesion layer, rather than the carrier particles, may dominate the cohesion. Thus, the layer-layer interlocking mechanism will be dominant even at low additions of fine drug, due to the reduced separation distance between the adhesive units. To understand the effect of fine particles on blend flowability, it is thus important to consider the degree of consolidation of the powder. The development of an increasingly voluminous adhesion layer will also create a more closed pore structure, i.e. a gradually decreasing pore diameter, which may explain the decreased permeability of the adhesive mixtures over a wide range of drug loads (Fig. 4).

### 3.4 Scales of flowability

The Hausner ratio and the flow function coefficient have been used to classify flowability into groups. The European Pharmacopoeia, version 9.0 (Council-of-Europe, 2017), reports a scale based on the compressibility of the powder, and another scale utilises the flow function coefficient (Jenike, 1964). In Table 1, the flow performance of the blends used here is presented using these scales.

**Table 1:** Classification of the flowability of adhesive mixtures using grading scales based on the Hausner ratio and the flowability function. Mean values (*n* = 3) and standard deviations.

Concentration API (%)		Inhalac 70				Inhalac 230		
	HR	Classification	Ffc	Classification	HR	Classification	Ffc	Classification
0.00	1.04 (0.00)	Excellent	9.67 (2.37)	Intermittently flowing				
0.50	1.06 (0.01)	Excellent	11.8 (2.31)	Free flowing				
1.00	1.05 (0.00)	Excellent	19.1 (2.05)	Free flowing				
1.51	1.05 (0.00)	Excellent	12.8 (7.92)	Free flowing				
2.02	1.05 (0.00)	Excellent	30.0 (12.2)	Free flowing				
4.04	1.07 (0.00)	Excellent	36.7 (2.06)	Free flowing				
6.06	1.09 (0.00)	Excellent	18.8 (3.30)	Free flowing				
8.08	1.10 (0.00)	Excellent	26.6 (7.13)	Free flowing				
10.10	1.12 (0.01)	Good	10.1 (3.46)	Free flowing				
14.14	1.14 (0.00)	Good	6.52 (0.01)	Intermittently flowing				
0.00					1.12 (0.00)	Good	7.94 (0.85)	Easy flowing
0.80					1.11 (0.00)	Excellent	9.94 (1.34)	Easy flowing
1.19					1.15 (0.01)	Good	11.1 (1.05)	Free flowing
2.38					1.17 (0.01)	Good	12.3 (4.31)	Free flowing
3.58					1.17 (0.02)	Good	8.81 (0.93)	Intermittently flowing
4.77					1.19 (0.02)	Fair	7.97 (1.87)	Intermittently flowing

The tests use different scale terms, but both indicate that the adhesive blends generally flow well. Although there is a lack of correlations between the compression and shear tests, they showed good agreement in their overall flow performance grading. The flow performance was more susceptible to the drug content for the Inhalac 230 blends than for the Inhalac 70 blends. Thus, the mass and morphology of the carrier influenced the effect of drug load on the flowability grading. For both carriers, the addition of the fine drug affected the grading of blend flowability, with the combination of carrier and adhesion layer properties dictating the flowability. The addition of fine particles decreased the flowability at the drug concentration at which free agglomerates were formed (blend state 3), i.e. at approximately 14 % fines for Inhalac 70 and at about 4 % fines for Inhalac 230. As pointed out earlier (Vasilenko et al., 2011), it is important that the method used to grade flowability is relevant to the intended application of the test results. However, several characterisation methods may be needed to provide a broad representation of the relevant flow properties of inhalation powders.

### 3.5 Relationships between mechanical properties and aerosolisation propensity

The aerosolisation propensity of the series of adhesive mixtures used here was presented earlier (Simonsson et al., 2024). A Next Generation Impactor (NGI) was used to assess mixture dispersion, and the parameters derived to quantify the dispersion were the fine particle fraction (FPF), using a cut-off diameter of 5 μm, and the median aerodynamic diameter (MMAD), calculated from the deposition of the drug in all cups of the impactor. Thus, the FPF represented only a fraction of deposited particles in the NGI. Two pressure drops (0.5 and 4 kPa) over the inhaler was used in the dispersion experiments, and it was observed that the effect of drug load on FPF and MMAD was dependent on the pressure drop. For Inhalac 70, an increased pressure drop tended to increase the fluctuations of FPF with drug load, while the converse applied for the MMAD. The evolution pattern of these fluctuations with drug load was described as consisting of two waves or valleys, i.e. a pattern similar to the changes observed in packing density and shearing for the Inhalac 70 mixtures (Fig. 6). Thus, we explored whether there was any correlation between the packing and flow properties of the mixtures, on one hand, and the dispersion properties, on the other. To this end, correlations were studied using Pearson analysis with FPF and MMAD data obtained at pressure drops of 0.5 kPa and 4 kPa. It should be noted that in these statistical tests, the variables for the carrier powder alone were excluded, as well as the highest drug loads used. The highest drug loads for the respective carrier were considered to represent segregated mixtures with two drug particle populations: one bonded within the adhesive units and one non-bonded, forming free drug agglomerates.

Generally, the *P*-values for the correlations between dispersion and mechanical parameters of the mixtures were generally higher than for the only mechanical properties discussed above, i.e. lower probabilities for correlations. This could partly be explained by the lower number of mixtures used in the statistical analysis.

**Fig. 8:**
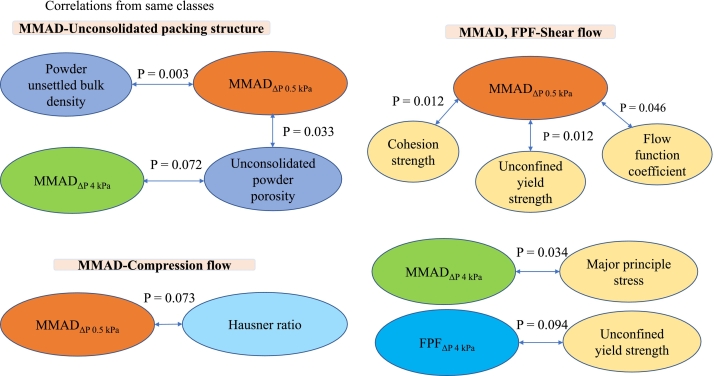
Correlations from the Pearson analysis between aerosolisation properties (FPF and MMAD) and test classes, as shown in Fig. 1, for all mixtures of Inhalac 70 (0.5–10 % Budesonide, *w*/w).

In Fig. 8, the correlations obtained for the Inhalac 70 mixtures are presented using a *P* < 0.10 as cut-off, with the respective P-values provided for the respective relationship. Some of the property-property relationships were significant, i.e. showing a P-value<0.05, while others show a weaker correlation (*P* = 0.05–0.10). For Inhalac 70, statistical significance was observed between MMAD and indications of both packing densities, shearing properties and compression, predominantly for MMAD assessed at a pressure drop of 0.5 kPa. The FPF, assessed at 4 kPa pressure drop, showed a weak tendency to correlate with the unconfined yield strength.

**Fig. 9:**
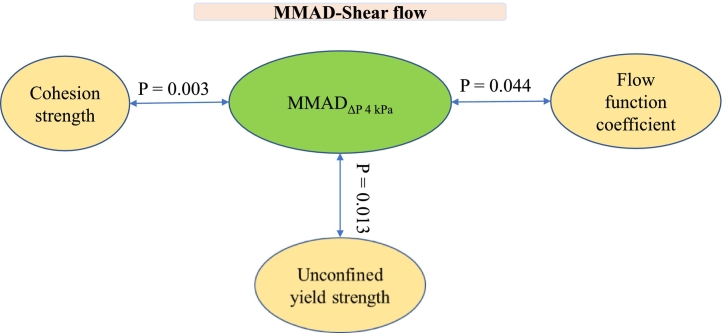
Correlations from the Pearson analysis between aerosolisation properties (FPF and MMAD) and test classes, as shown in Fig. 1, for all mixtures of Inhalac 230 (0.8–3.58 % Budesonide, w/w).

In Fig. 9, the correlations obtained for the Inhalac 230 mixtures are presented. Statistically significant relationships were observed for MMAD assessed at a pressure drop of 4 kPa, along with some indicators of the shearing properties of the blends.

The dispersion of the adhesive mixture can be somewhat simplified as consisting of two processes, i.e. the liberation (detachment) of primary drug particles or micro-agglomerates thereof and the disintegration of these micro-agglomerates as they are transported into the impactor. Important mechanisms causing detachment are suggested to include (Kassinos et al., 2021, p. 125–126) spinning, collision and shearing/erosion. The packing and shearing of a powder, on the other hand, are dictated by the interactions between the particles (the adhesive units). Therefore, dispersion and flow are fundamentally different properties of adhesive mixtures. Moreover, both properties can be assessed in a multitude of ways, depending on the experimental conditions. Thus, using flow properties as a general means to predict dispersion is questionable. Nevertheless, significant correlations have been presented in the literature between these blend properties, (referring to the selection of studies in section 1), due to the selection of certain experimental conditions.

**Fig. 10:**
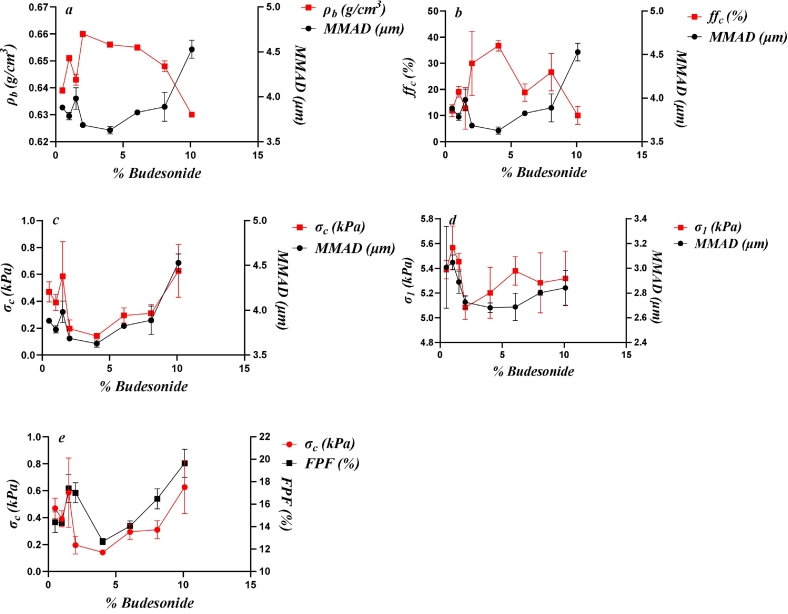
The effect of drug load on drug load on aerosolisation and selected properties, as shown in Fig. 1, for all mixtures of Inhalac 70 (% Budesonide 0.5–10 % w/w). Mean values and standard deviations (n = 3). (a) Bulkdensity and MMAD (ΔP 0.5 kPa). (b) Flow function coefficient and MMAD (ΔP 0.5 kPa). (c) Unconfined yield strength and MMAD (ΔP 0.5 kPa (d) Major principal stress and MAMD (ΔP 4 kPa). (e) Unconfined yield strength and FPF (ΔP 4 kPa).

**Fig. 11:**
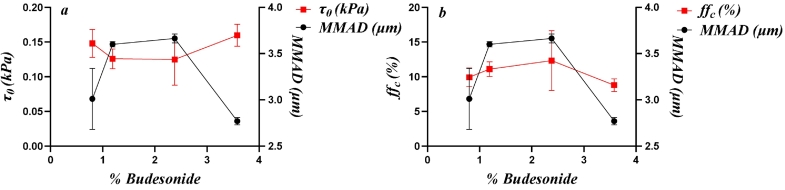
The effect of drug load on drug load on aerosolisation and selected properties, as shown in Fig. 1, for all mixtures of Inhalac 230 (% Budesonide 0.8–3.58 % w/w). Mean values and standard deviations (n = 3). (a) Cohesion strength and MMAD (ΔP 4 kPa). (b) Flow function coefficient and MMAD (ΔP 4 kPa).

In this study, complex blend property-drug load patterns were observed for the packing and shearing properties of the mixtures under unconfined conditions. Qualitatively, these relationships are similar to the blend dispersion–drug load relationships reported earlier (Simonsson et al., 2024). In Fig. 10 (Inhalac 70 mixtures) and Fig. 11 (Inhalac 230 mixtures), the dispersion parameters showing the highest probabilities for correlation with the mechanical parameters are plotted on the same graph. The evolution patterns of both the dispersion and mechanical parameters showed obvious conformity, and the respective blend property-drug load relationships could be described as nearly parallel. For Inhalac 70 mixtures, both the dispersion and the mechanical parameters had two waves in their progression, while for Inhalac 230 mixtures, a single wave was obtained.

The underlying cause of the co-variation in blend properties with drug load is proposed to be a similar dependency on the blend architecture and the structure of the adhesion layer. Thus, these blend structural features are critical for both the dispersion and mechanical properties of an adhesive mixture. Any change in the architecture of an adhesive mixture, for example, due to changed particle properties (Kalman and Portnikov, 2021) or changes in mixing conditions, can hence potentially affect the packing, flow and dispersion properties of the mixture simultaneously.

**Fig. 12:**
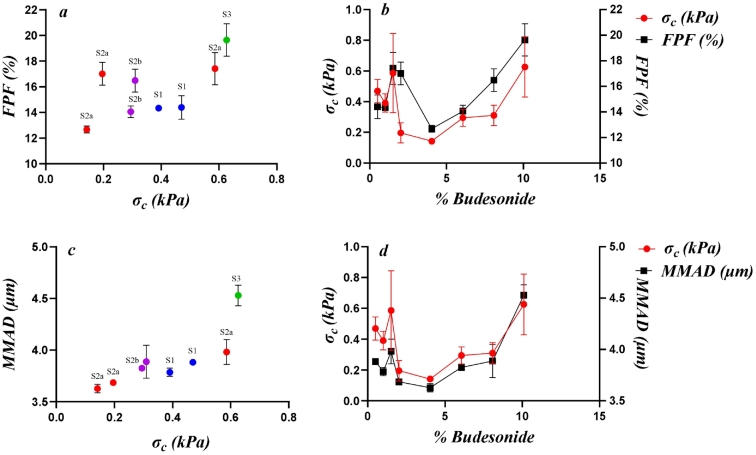
The effect of drug load and unconfined yield strength on FPF (ΔP 4 kPa) and MMAD (ΔP 0.5 kPa). Mean values and standard deviations (n = 3). (a,c) The effect of unconfined yield strength on FPF and MMAD, and the positions of the corresponding blend states (Simonsson et al., 2024). (b,d) The effect of drug load on unconfined yield strength, FPF and MMAD.

In Fig. 12, the MMAD and FPF data with the highest probabilities of correlating to the unconfined yield strength (see Fig. 10) are plotted for the Inhalac 70 mixtures. In the graphs, the data points proposed to be related to the different blend states, as determined earlier (Simonsson et al., 2024), are marked. The general trend observed is that an increased unconfined yield strength, i.e. increased internal friction in the mixtures, corresponded to an increased FPF and increased MMAD. Due to the fluctuations in the blend property-drug load relationships, the blend states did not follow a sequential order in the MMAD vs unconfined yield strength relationship, but these properties were nearly linearly related to each other. The suggested mechanisms explaining the shear strength-FPF correlations are summarised in Table 2.

**Table 2:** Proposed relative effects of drug concentration, condensed into blend states, on the dispersion and cohesion of binary adhesive mixtures, explaining the correlation between these blend properties.

State	Fines localisation	Degree of dispersion	Unconsolidated cohesion strength
1a	Adsorbed as singular particles in surface cavities	High FPF due to inertial detachment of single particles	Low due to the increased carrier-to-carrier separation distance caused by singular adsorbed particles in cavities, resulting in decreased carrier-to-carrier cohesion
1b	Adsorbed as a multilayer of particles in surface cavities	Low FPF due to inertial detachment of micro-agglomerates that are not completely disintegrated	High due to increased friction (mechanical interlocking) between a multilayer of particles in cavities and carrier asperities
2a	Adsorbed as singular particles on the enveloped surface	High FPF due to inertial and erosion detachment of single particles, followed by a gradual decrease in FPF caused by the formation of a saturated monolayer	Low due to a further increase in carrier-to-carrier separation distance caused by singular adsorbed particles on the enveloped surface, leading to decreased carrier-to-carrier cohesion
2b	Adsorbed as a multilayer of particles on the enveloped surface	Low FPF due to erosion detachment of micro-agglomerates, followed by a gradual increase in FPF as the adhesion layer becomes more and more voluminous	Intermediate due to increased friction (mechanical interlocking) between adhesion units, due to the uneven texture of a voluminous adhesion layer
3	Multilayer of particles bonded in adhesive units, with non-bonded particles present as free agglomerates	High FPF due to erosion detachment of micro-agglomerates and the disintegration of free agglomerates	Very high due to significant friction between adhesion units and free-agglomerates

## 4 Conclusion

In the formulation of adhesive mixtures for inhalation, the relationship between dispersion and the mechanical (packing and flow) properties of the mixture needs to be considered. In this study, we investigated potential correlations between the packing and flow properties of binary adhesive mixtures consisting of micronised budesonide (as a model drug) and a conventional lactose carrier. The study was conducted with the overall goal of maximising the drug load, i.e. at which state 3 is reached. Thus, a broad range of drug loads was used. The key findings of this study are as follows:

• The test method used to assess mechanical properties influences the property-drug load relationship. The test methods could be classified into two groups: packing density and shearing properties belonged to one group, and permeability and compressibility formed the other.
• The shearing tests indicated that the fine drug particles can act both as a glidant, reducing the contact area between the carriers, and as an anti-glidant, increasing the friction between the adhesive units.
• Previously published FPF and MMAD data (Simonsson et al., 2024) correlated with the shearing and packing properties in an unconfined state, while permeability, which was dependent on the blend pore diameter, did not co-vary with the blend dispersibility. The proposed explanation for this observed co-variation is a similar dependency of the blend architecture and the structure of the adhesion layer.
• Regarding the effect of blend state on mixture properties, blend state 2a provided an optimal balance between dispersibility (a high FPF and a low MMAD) and flowability (low cohesion strength).

Important applications of these findings in the formulation of adhesive mixtures for inhalation include the following:

• It is preferable to use several methods to assess the packing and flow properties of adhesive mixtures rather than relying on a single method. The selected test methods should represent different categories of tests. To predict the mixture dispersibility based on mechanical testing, a validated test method that has demonstrated a strong correlation with dispersibility should be used. In order to find the optimal mixture performance from a broad perspective, four key performance aspects of adhesive mixtures must be considered. These aspects, which may all depend on the blend state, include dispersibility, packing density, flowability and segregation propensity.
• Any change in the architecture or state of an adhesive mixture can simultaneously affect both the mechanical and dispersion properties of the mixture, thereby influencing the performance of the inhalation powder during manufacturing and use. Blend architecture may thus be a key aspect to understand and consider during the formulation and engineering of adhesive mixtures for optimal performance. Changes in formulation, e.g. variation in particle properties, the addition of a ternary component or changes to mixing conditions, may ultimately lead to changes in blend architecture and state, predominantly in the structure of the adhesion layer.

It should be pointed out that the relationships studied here should also be investigated for inhalers used in commercial drug product development. First, during manufacturing of inhalation powders, the filling of the powder into the inhaler may require a densification of the powder and the powders are thus in a slightly consolidated state in contrast to the unconsolidated state used in this study. The consolidation may affect both the mechanical properties and also the dispersion of the powder bed, e.g. due to a change in the structure of the adhesion layer. Possible consolidation effects on the adhesion layer are a redistribution of fines from enveloped surfaces to cavities and the compression of a voluminous adhesion layer. The consequent effects on property-drug load relationships of such changes need to be studied but one can speculate that the effects are more pronounced at high drug loads at which the adhesion layer is more susceptible to physical changes. Secondly, the experimental inhaler used here may not be representative for commercial inhalers since it is a simple construction which can be expected to have a limited powder retention and a relatively low dispersion capacity. Commercial inhalers have a more complicated geometry and a higher inhaler resistance which can affect the retained dose and the degree dispersion of drug in the inhaler. Thus, for commercial inhalers, relationships between mechanical and aerosolization properties need to be experimentally studied. It is however hypothesized that correlations similar to reported here can be assumed mediated by the structure of the adhesion layer.

## CRediT authorship contribution statement

**Anna Simonsson:** Writing – review & editing, Writing – original draft, Validation, Methodology, Investigation, Formal analysis, Conceptualization. **Tobias Bramer:** Writing – review & editing, Supervision, Resources, Conceptualization. **Alex Wimbush:** Writing – review & editing, Supervision, Conceptualization. **Göran Alderborn:** Writing – review & editing, Writing – original draft, Supervision, Resources, Methodology, Funding acquisition, Conceptualization.

## Declaration of competing interest

The authors declare the following financial interests/personal relationships which may be considered as potential competing interests: Anna Simonsson reports financial support was provided by Sweden's Innovation Agency. Tobias Bramer, Alex Wimbush reports financial support and equipment, drugs, or supplies were provided by AstraZeneca R&D Gothenburg. Tobias Bramer, Alex Wimbush reports a relationship with AstraZeneca R&D Gothenburg that includes: employment. Göran Alderborn is an editorial board member at Int. J. Pharm If there are other authors, they declare that they have no known competing financial interests or personal relationships that could have appeared to influence the work reported in this paper.

## Data Availability

Data will be made available on request.
